# POCUS-Guided Forearm Nerve Blocks for Analgesia of Acute Hand Ischemia in the Emergency Department

**DOI:** 10.24908/pocusj.v10i02.19734

**Published:** 2025-11-17

**Authors:** Richard J. Gawel, Sydney Sillart, Nova Panebianco, Michael Shalaby, Ian P. Ciesielski

**Affiliations:** 1 Department of Emergency Medicine, Hospital of the University of Pennsylvania, Philadelphia, Pennsylvania, USA; 2 Division of Emergency Ultrasound, Hospital of the University of Pennsylvania, Philadelphia, Pennsylvania, USA

**Keywords:** Regional anesthesia, Pain Management, Upper extremity ischemia, Point-of-care ultrasound guided regional anesthesia

## Abstract

**Background:**

Acute limb ischemia (ALI) is a vascular emergency associated with significant pain that can be challenging to manage, especially in opioid-tolerant patients.

**Case Report:**

A 44-year-old man with opioid use disorder presented with ALI of the right hand not amenable to surgical intervention after self-injection of fentanyl. Despite high-dose opioids, he continued to experience refractory pain. Point of care ultrasound (POCUS)-guided radial and median nerve blocks performed in the emergency department provided substantial relief.

**Discussion:**

This case illustrates the novel use of POCUS-guided upper extremity regional anesthesia by emergency physicians to manage ALI pain. POCUS-guided regional anesthesia may be a safe, effective adjunct in select patients, though patients must be closely monitored for complications.

## Introduction

Acute limb ischemia (ALI) results from a sudden and critical decrease in blood supply to an extremity. Although relatively uncommon, ALI carries considerable morbidity and risk of limb loss [1,2]. The most frequent cause is embolic occlusion in patients with underlying peripheral arteriosclerosis, though in rare cases ALI can result from external compression of the artery from surrounding soft tissue inflammation [3]. Clinically, patients with ALI typically present with severe pain, paresthesia, weakness, and discoloration of the affected limb [3,4]. Without proper treatment, ALI can result in distal limb necrosis and limb loss [3,4].

Pain associated with ALI can be profound, especially during the active phase of ischemia, and may be challenging to manage. For cases of ALI not amenable to surgical intervention, management typically includes anticoagulation and analgesics [5]. We report the use of point of care ultrasound (POCUS)-guided regional anesthesia performed by emergency physicians in the emergency department (ED) to manage pain associated with non-operative ALI of the hand.

## Case Report

**Figure 1.**
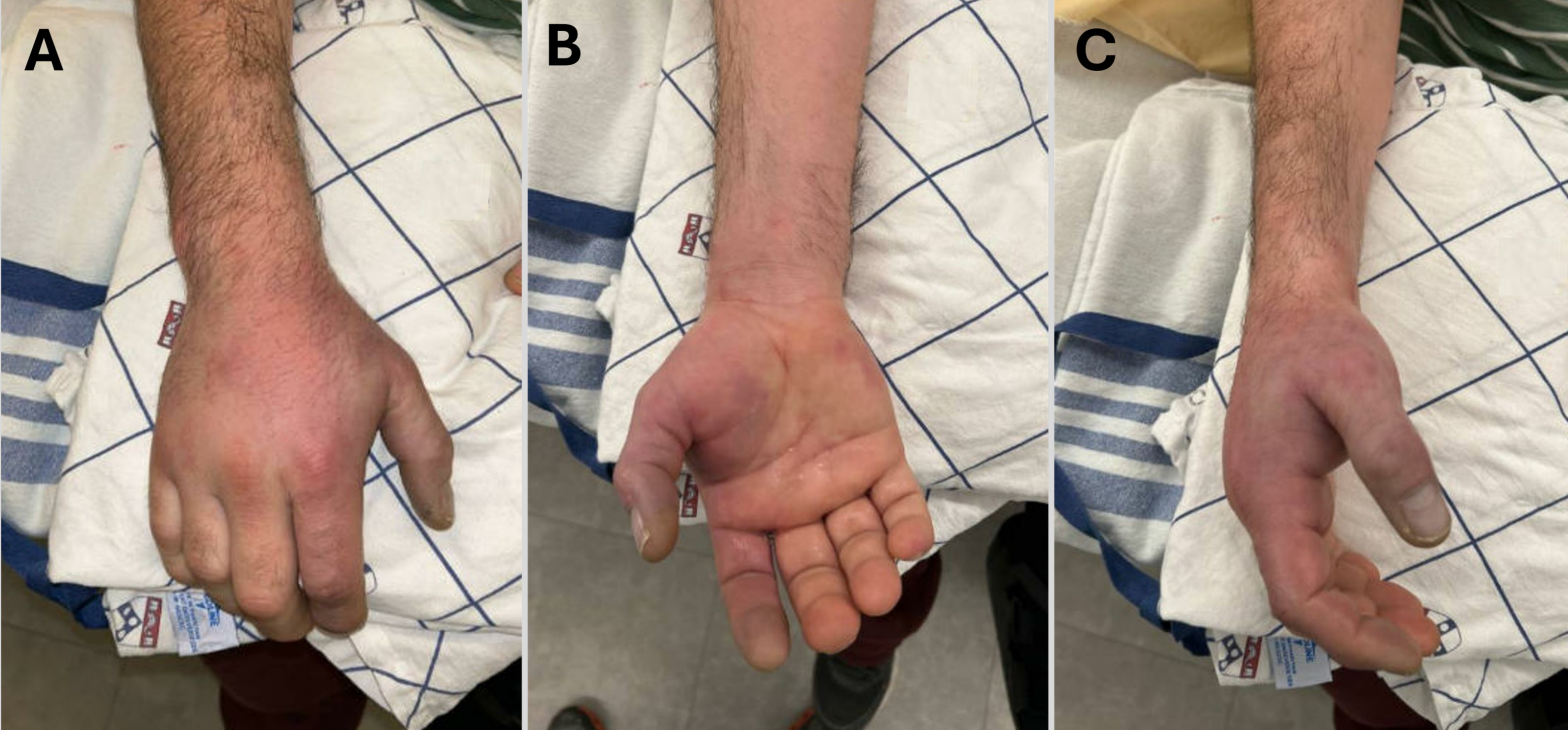
Images of the right hand in pronated (Fig 1A), supinated (Fig 1B), and neutral (Fig 1C) position illustrating the erythematous and dusky appearance of the first and second fingers and the radial surface of the hand resulting from acute occlusion of the distal radial artery at the level of the wrist.

A 44-year-old man with opioid use disorder presented to the ED with worsening pain, swelling, numbness, and dusky discoloration of his right hand and fingers, five days after injecting fentanyl into the volar aspect of his right wrist. He was hemodynamically stable and afebrile but in significant pain. Physical exam revealed dusky discoloration of the first and second digits, tenderness along the dorsal right hand and distal forearm, decreased sensation to pinprick, and weakness of the first and second digits. The hand compartments were soft (Figure 1A-C).

Laboratory testing revealed a mildly elevated creatinine kinase (3,909 U/L) without leukocytosis, lactic acidosis, anemia, or electrolyte abnormalities. A computed tomography angiogram of the right upper extremity demonstrated an acute occlusion of the distal right radial artery at the wrist, just distal to the takeoff of the superficial palmar arch. Surgical consultation determined that findings were due to acute inflammation from caustic materials in the fentanyl injection rather than a true arterial obstruction and recommended medical management. The patient was started on a heparin infusion titrated to a partial thromboplastin time of 60 to 85 seconds and was admitted for serial compartment checks.

**Figure 2.**
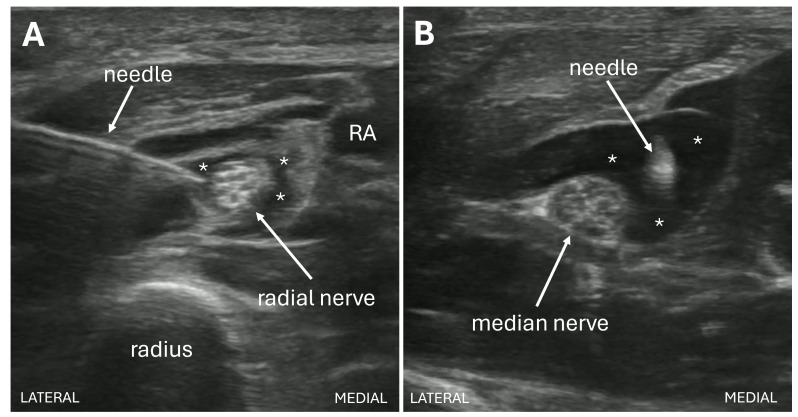
Point of care ultrasound (POCUS) images of radial (Fig 2A) and medial (Fig 2B) nerve block performed at the level of the mid forearm under real time POCUS guidance. Figure 2A shows the radial nerve block performed using in-plane needle visualization, with anesthetic deposited around the radial nerve just lateral to the radial artery (RA). Figure 2B shows the median nerve block performed using out-of-plane needle visualization, showing the needle tip within the surrounding anesthetic. The asterisks (*) signify the hypoechoic anesthetic in both blocks.

While boarding in the ED, the patient continued to experience severe pain despite high-dose multimodal analgesia, including a hydromorphone patient-controlled analgesia (PCA, 0.6 mg demand dose every 10 minutes) that was initiated to manage opioid withdrawal. He subsequently underwent POCUS-guided radial and median nerve blocks at the level of the mid-forearm with 5 mL of 0.2% ropivacaine administered around each nerve (Figures 2A–B). Both blocks were performed by a post-graduate year 3 (PGY-3) emergency medicine resident who had previously performed more than 30 POCUS-guided nerve blocks under the supervision of an ultrasound fellowship-trained attending physician who had performed or supervised more than 40 POCUS-guided nerve blocks. At reassessment 60 minutes after the blocks, the patient's pain improved from 10/10 to 2/10 on the Numeric Rating Scale (NRS). No immediate complications were encountered.

This degree of pain relief was maintained for approximately 16 hours, after which he experienced a gradual return of discomfort with documented NRS scores ranging from 3/10 to 5/10 over the next several days, representing an improvement compared to pre-block pain levels. Due to his high baseline opioid tolerance and active withdrawal, he continued to receive systemic opioids via the hydromorphone PCA immediately after the block. By the following day, his PCA was discontinued, and he was transitioned to oral opioids. He continued to require substantial opioid therapy for withdrawal management, though his hand pain remained well-controlled. One week into his hospitalization, he developed a multicompartment abscess at the fentanyl injection site requiring several surgical debridements, each performed under a continuous supraclavicular brachial plexus block catheter placed by an anesthesiologist. He was ultimately transitioned to a direct oral anticoagulant and discharged with follow-up arranged with hand surgery. The patient provided consent that his deidentified information and case images be used for the purposes of publication.

## Discussion

We describe the successful use of POCUS-guided radial and medial nerve blocks for a patient in the ED with severe opioid-refractory pain from caustic compression of his radial artery that was not amenable to surgical intervention. To our knowledge, this is the first reported case of POCUS-guided regional anesthesia performed by emergency physicians to manage pain from upper extremity ALI.

Regional nerve blocks have been used in the perioperative and critical care setting to facilitate pain management for ALI [6,7]. While one prior case described the use of combined popliteal-sciatic and saphenous nerve blocks for lower extremity ALI awaiting urgent thrombectomy, our case was unique in that surgical intervention was not indicated [8]. As a result, the patient was expected to endure severe pain for several days during the initial phase of infarction of his hand. Moreover, his high opioid tolerance limited the effectiveness of opioid analgesia for acute pain management. For these reasons, multimodal analgesia, including POCUS-guided regional anesthesia, was an ideal approach for pain control in this case.

In addition to the usual risks of POCUS-guided regional anesthesia, its use in ALI carries the added concern of potentially masking the development of compartment syndrome, which is a known complication of ALI [9]. Recent literature, however, suggests that with careful monitoring, nerve blocks can still be used safely even in patients at risk for compartment syndrome [10–13]. In this case, our patient was admitted and closely followed by hand surgery for frequent neurovascular and compartment checks to monitor any developing signs or symptoms of compartment syndrome. Given the nature of this case, effective communication between the emergency physician performing the block and the surgical consultant and inpatient providers was essential to facilitate a safe transition of care.

In conclusion, this case highlighted a novel application of POCUS-guided upper extremity regional anesthesia by emergency physicians to manage severe ALI pain. Further studies are needed to evaluate the safety and efficacy of this approach in larger patient cohorts. As always, close monitoring is essential to detect potential complications, and decisions to use regional anesthesia in ALI should be made collaboratively with surgical and inpatient teams.
